# The Chemotaxonomy of Common Sage (*Salvia officinalis*) Based on the Volatile Constituents

**DOI:** 10.3390/medicines4030047

**Published:** 2017-06-29

**Authors:** Jonathan D. Craft, Prabodh Satyal, William N. Setzer

**Affiliations:** Department of Chemistry, University of Alabama in Huntsville, Huntsville, AL 35899, USA; jdc0060@uah.edu (J.D.C.); prabodhsatyal@gmail.com (P.S.)

**Keywords:** sage oil, chemical composition, cluster analysis

## Abstract

**Background:** Common sage (*Salvia officinalis*) is a popular culinary and medicinal herb. A literature survey has revealed that sage oils can vary widely in their chemical compositions. The purpose of this study was to examine sage essential oil from different sources/origins and to define the possible chemotypes of sage oil. **Methods:** Three different samples of sage leaf essential oil have been obtained and analyzed by GC-MS and GC-FID. A hierarchical cluster analysis was carried out on 185 sage oil compositions reported in the literature as well as the three samples in this study. **Results:** The major components of the three sage oils were the oxygenated monoterpenoids α-thujone (17.2–27.4%), 1,8-cineole (11.9–26.9%), and camphor (12.8–21.4%). The cluster analysis revealed five major chemotypes of sage oil, with the most common being a α-thujone > camphor > 1,8-cineole chemotype, of which the three samples in this study belong. The other chemotypes are an α-humulene-rich chemotype, a β-thujone-rich chemotype, a 1,8-cineole/camphor chemotype, and a sclareol/α-thujone chemotype. **Conclusions:** Most sage oils belonged to the “typical”, α-thujone > camphor > 1,8-cineole, chemotype, but the essential oil compositions do vary widely and may have a profound effect on flavor and fragrance profiles as well as biological activities. There are currently no studies correlating sage oil composition with fragrance descriptions or with biological activities.

## 1. Introduction

Sage (also known as garden sage, common sage, or culinary sage; *Salvia officinalis* L., Lamiaceae) is a popular culinary and medicinal herb, native to southern Europe and the Mediterranean, but now cultivated worldwide. The plant has been used since ancient times for various human ailments. For example, in England, a decoction of sage leaves with wine was gargled to relieve toothache [1]; in Germany, sage was used orally for gastrointestinal problems and excessive perspiration, and was used topically for inflammation of the mucous membranes of the mouth and throat [2]; the Cherokee Native Americans have used an infusion of the plant to treat colds and coughs, and as an antidiarrheal [3]. Commercial sage oil is generally characterized by thujones, with α-thujone usually predominating (18–43%) over β-thujone (3–8.5%), camphor (4.5–24.5%), 1,8-cineole (5.5–13%), α-humulene (0–12%), α-pinene (1–6.5%), camphene (1.5–7%), and bornyl acetate (2.5% maximum) [2].

Caution should be exercised in using sage essential oil. The oil contains large concentrations of α-thujone, which was thought to have been the hallucinogenic constituent of absinthe and the cause of absinthism. This, however, has been shown to be false [4]. Nevertheless, high doses of α-thujone causes convulsions by way of blocking γ-aminobutyric acid (GABA)-gated chloride channels [5,6], and chronic exposure can lead to neurotoxicity [7,8] and carcinogenicity [9]. Use of the herb itself is safe, however; it has been estimated that between 2 and 20 cups of sage tea would be required to reach the acceptable daily intake of thujone [10]. Additionally, thujone has shown a low affinity for cannabinoid receptors, but failed to show cannabinoid receptor agonism [11]. α-Thujone has also been shown to reduce 5-HT_3_ (ligand-gated ion channel serotonin) receptor activity [12]. In this work, we have characterized two commercial sage essential oils as well as an essential oil obtained by hydrodistillation of sage leaves grown in Mexico. In addition, a cluster analysis has been carried out to place the different chemotypes of sage oil in perspective.

## 2. Materials and Methods

### 2.1. Essential Oils

Fresh sage (*Salvia officinalis*, Jacobs Farm organic sage, Pescadero, CA, USA, grown in Mexico) was purchased from a local market in Huntsville, Alabama on 8 April 2017. The fresh leaves (34.64 g) were chopped and hydrodistilled using a Likens–Nickerson apparatus for 4 h with continuous extraction with dichloromethane (CH_2_Cl_2_) to give 1.653 g yellow essential oil. Commercial sage leaf essential oils were obtained from Mountain Rose Herbs (Eugene, OR; oil from California) and Selikaj Ltd. (Koplik, Albania).

### 2.2. Gas Chromatography-Mass Spectrometry

The leaf essential oil samples of *Salvia officinalis* were analyzed by GC-MS using an Agilent 6890 gas chromatograph coupled to an Agilent 5973 mass selective detector (MSD), operated in the electron impact mode with electron energy = 70 eV, a scan range of 40–400 amu, a scan rate of 3.99 scans/sec, and operated through an Agilent ChemStation data system. The GC column was an HP-5 ms fused silica capillary column with a (5% phenyl)-polydimethylsiloxane stationary phase, a film thickness of 0.25 μm, a length of 30 m, and an internal diameter of 0.25 mm. The carrier gas was helium with a column head pressure of 92.4 kPa and a flow rate of 1.5 mL/min. The inlet temperature was 250 °C and the interface temperature was 280 °C. The GC oven temperature was programmed, 60 °C initial temperature, which was held for 5 min, temperature increased at a rate of 3 °C/min up to 280 °C. Solutions of essential oils (1% in CH_2_Cl_2_) were prepared and 1-μL injections were carried out using a splitless mode. Identification of the oil components was based on their retention indices determined by reference to a homologous series of *n*-alkanes, and by comparison of their mass spectral fragmentation patterns with those reported in the literature [13], and stored in our in-house MS library.

### 2.3. Quantitative Gas Chromatography

Quantitative GC was carried out using an Agilent 6890 GC with Agilent flame ionization detector (FID), HP-5ms column, helium carrier gas (head pressure = 144.1 kPa, flow rate = 2.0 mL/min), same oven temperature program as GC-MS (above). The percentages of each component in the essential oils are reported as raw percentages without standardization.

### 2.4. Hierarchical Cluster Analysis

A total of 185 *S. officinalis* leaf essential oil compositions from the published literature [14,15,16,17,18,19,20,21,22,23,24,25,26,27,28,29,30,31,32,33,34,35,36,37,38,39,40,41,42,43,44,45,46,47,48,49,50,51,52,53,54,55,56,57,58,59,60,61,62,63,64,65,66], as well as the three compositions from this study were treated as operational taxonomic units (OTUs). The percentage composition of 26 major essential oil components (α-pinene, camphene β-pinene, myrcene, α-phellandrene, *p*-cymene, limonene, 1,8-cineole, (*E*)-β-ocimene, γ-terpinene, α-thujone, β-thujone, camphor, borneol, α-terpineol, bornyl acetate, α-terpinyl acetate, β-caryophyllene, aromadendrene, α-humulene, viridiflorene, viridiflorol, humulene epoxide II, pimaradiene, manool, and sclareol) was used to determine the compositional associations of the various *S. officinalis* essential oil samples by agglomerative hierarchical cluster (AHC) analysis using the XLSTAT software, version 2015.4.01. Pearson correlation was selected as a measure of similarity, and the unweighted pair-group method with arithmetic average (UPGMA) was used for cluster definition.

## 3. Results and Discussion

The sage leaf essential oil compositions are summarized in Table 1. The sage oils were qualitatively similar and dominated by the monoterpenoids α-thujone (17–27%), 1,8-cineole (12–27%), and camphor (13–21%), with lesser amounts of β-thujone (3.8–6.0%), camphene (3.5–5.3%), and the sesquiterpene α-humulene (3.1–4.4%). This chemical profile is similar to many sage oil descriptions previously reported [15,17,18,19,20,22,24,25,26,27,28,29,30,31,32,33,37,39,40,41,42,44,45,47,49,51,52,53,55,57,58,60,61,63,64,65,67], yet notably different from many others [14,25,26,34,37,38,41,46,54,56]. This prompted us to undertake a hierarchical cluster analysis of *S. officinalis* leaf oil compositions in order to describe the various chemotypes of this herb.

Tucker and Maciarello described five groups based on four principal constituents: (1) camphor > α-thujone > 1,8-cineole > β-thujone; (2) camphor > α-thujone > β-thujone > 1,8-cineole; (3) β-thujone > camphor > 1,8-cineole > α-thujone; (4) 1,8-cineole > camphor > α-thujone > β-thujone; and (5) α-thujone > camphor > β-thujone > 1,8-cineole [61]. Unfortunately, while these four principal constituents describe many *S. officinalis* essential oils, there are other samples that are rich in α-humulene [41,56], viridiflorol [26,34], manool [34,66], or sclareol [54].

Jug-Dujaković and co-workers examined the essential oil compositions of 25 indigenous populations of *S. officinalis* growing in the Dalmatian region of Croatia [37]. These workers carried out a hierarchical cluster analysis based on eight principal components (α-thujone, camphor, β-thujone, 1,8-cineole, β-pinene, camphene, borneol, and bornyl acetate), and were able to delineate three chemotypes of Dalmatian sage from Dalmatia: (A) α-thujone > camphor > 1,8-cineole > β-thujone; (B) β-thujone > α-thujone > camphor ≈ 1,8-cineole; and (C) camphor > α-thujone > 1,8-cineole > camphene ≈ borneol.

Lakušić and co-workers analyzed *S. officinalis* essential oils in various stages of development [41]. These workers sampled two different individual plants from different geographical origin, but grown in a common garden under identical conditions. Young leaves were characterized with high concentrations of α-humulene, viridiflorol, and manool, but low concentrations of camphor or α-thujone. As leaves aged, the concentrations of α-humulene, viridiflorol, and manool dropped significantly with concomitant increases in camphor and α-thujone. A hierarchical cluster analysis showed that young leaves belonged to an α-humulene chemotype, while old leaves from the plant originating in Serbia belonged to a camphor chemotype, and old leaves from the plant originating in Croatia belonged to a thujone chemotype.

**Figure 1:**
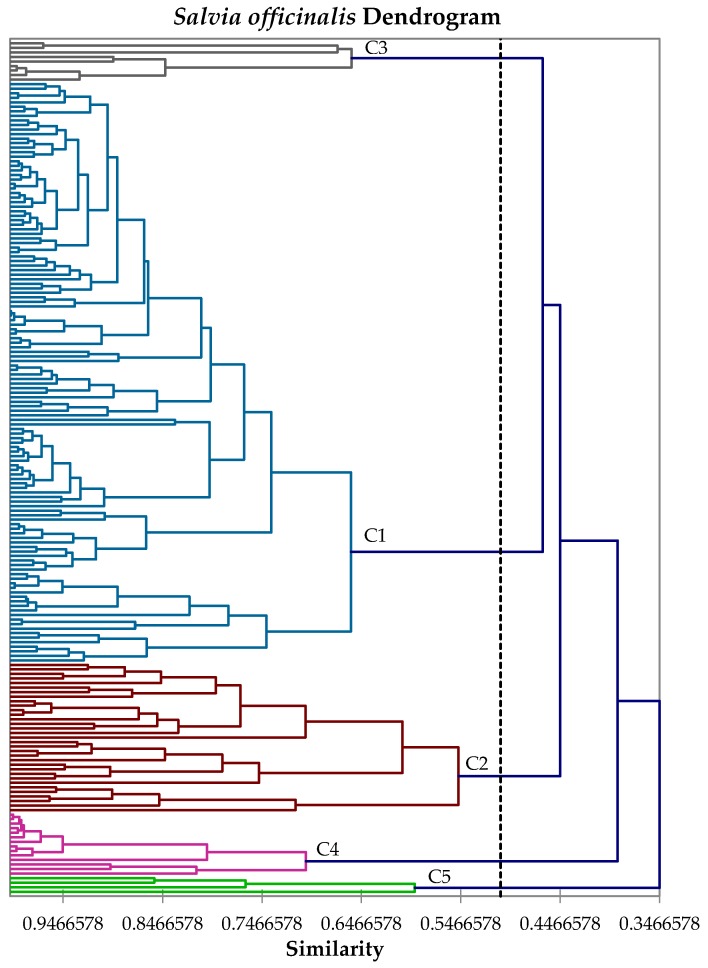
Dendrogram obtained from the agglomerative hierarchical cluster analysis of 188 *Salvia officinalis* leaf essential oil compositions. (C1) α-thujone/camphor chemotype, (C2) α-humulene/α-thujone chemotype, (C3) β-thujone/α-thujone/camphor chemotype, (C4) 1,8-cineole/camphor chemotype, and (C5) sclareol/α-thujone chemotype.

In this current work, we have carried out a hierarchical cluster analysis of 188 *S. officinalis* leaf essential oil compositions; the three chemical compositions presented above in conjunction with 185 analyses from the literature. A total of 26 components were used in the analysis. Based on the cluster analysis of the volatile compositions, there are five major chemotypes of *Salvia officinalis*: C1–C5 (see Figure 1).

**Figure 2:**
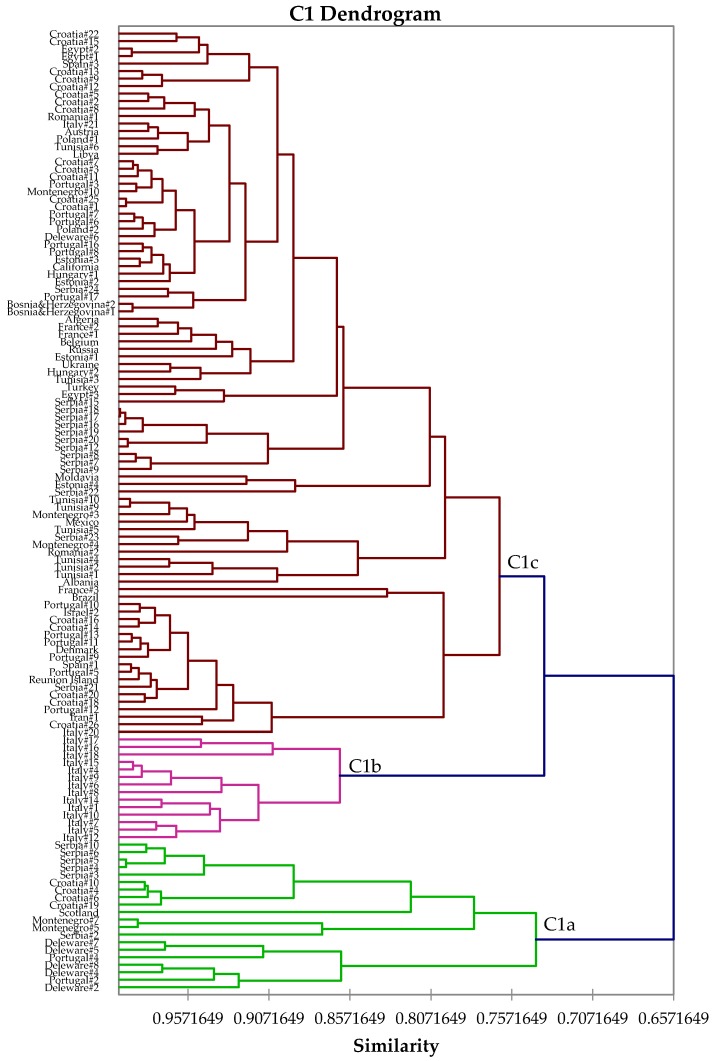
Expanded view of the dendrogram of C1 (α-thujone/camphor) chemotype.

The most populated chemotype, C1, is an α-thujone/camphor chemotype and represents “typical” sage oil. The C1 cluster can be further subdivided (Figure 2) into three distinct subgroups: C1a, camphor > α-thujone > β-pinene, which is equivalent to group 1 described by Tucker and Maciarello [61], type C described by Jug-Dujaković et al. [37], and type IIb described by Lakušić and co-workers [41]; C1b, α-thujone ≈ camphor > sclareol; and C1c, α-thujone > camphor > 1,8-cineole, which is equivalent to Tucker and Maciarello type 5 [61], Jug-Dujaković et al. type A [37], and Lakušić et al. type IIa [41]. Chemotype C1c averages 28.0% α-thujone, 18.6% camphor, 10.5% 1,8-cineole, and 6.4% β-thujone, and represents the “best overall” composition of sage oil [2,61]. It is noteworthy that type C1c is also represented by samples from the Dalmatian region of the Balkan Peninsula [26,37,41], as well as commercial samples from Europe [52] and Albania, Mexico, and California from this study.

**Figure 3:**
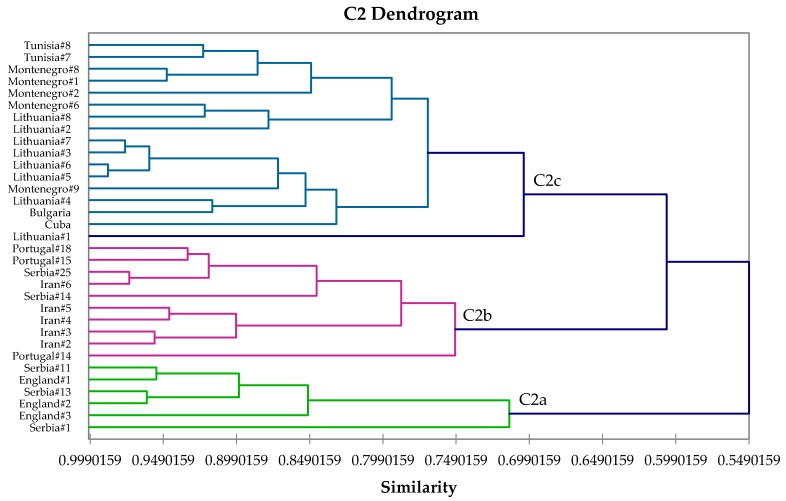
Expanded view of the dendrogram of C2 (α-humulene/α-thujone) chemotype.

The α-humulene-rich chemotype, C2, is equivalent to type I that was described by Lakušić and co-workers [41]. This chemotype can be subdivided (Figure 3) into three subgroups: C2a, α-humulene > α-thujone > camphor; C2b, 1,8-cineole ≈ α-thujone > α-humulene; and C2c, viridiflorol > manool ≈ α-thujone > α-humulene. Lakušić and co-workers had observed α-humulene concentrations to be relatively high in young leaves collected in April and May, with decreasing concentrations during late summer (August–October), and then increasing again in the autumn and winter [41]. Samples from other global locations, however, showed high α-humulene concentrations during the summer [26,48,56], and likely, then, represents a real chemotype.

**Figure 4:**
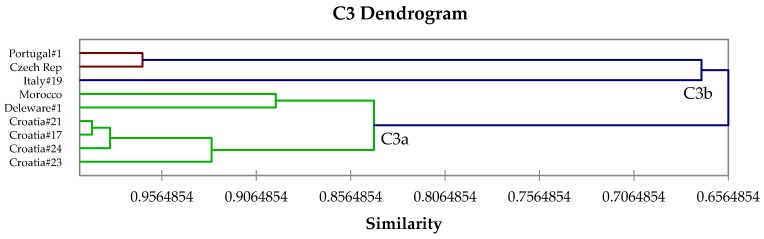
Expanded view of the dendrogram of C3 (β-thujone/α-thujone/camphor) chemotype.

**Figure 5:**
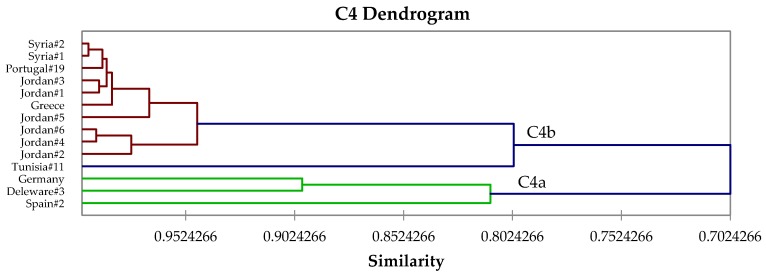
Expanded view of the dendrogram of C4 (1,8-cineole/camphor) chemotype.

**Figure 6:**
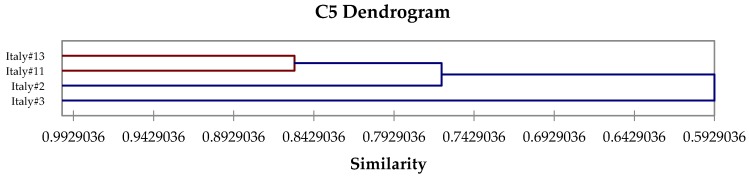
Expanded view of the dendrogram of C5 (sclareol/α-thujone) chemotype.

The β-thujone-rich chemotype, C3, is equivalent to Tucker and Maciarello type 3 [61] and Jug-Dujaković et al. type B [37]. Chemotype C3 can be subdivided into two subroups (Figure 4): C3a, β-thujone > camphor ≈ α-thujone ≈ 1,8-cineole, and C3b, camphor > β-thujone > 1,8-cineole. Type C4, a 1,8-cineole/camphor chemotype, is equivalent to Tucker and Maciarello type 4 [61], and shows two subtypes: C4a, 1,8-cineole ≈ camphor, and C4b, 1,8-cineole >> camphor (Figure 5). Chemotype C5 (Figure 6) is a sclareol/α-thujone type.

Although C1 (thujone/camphor) is the major chemotype of *S. officinalis*, there are several other chemotypes and this should have a profound effect on the flavor and fragrance profile of the herb as well as any potential biological activities and medicinal uses. The overall fragrance description and the fragrance descriptions of the components of C1c type sage oils have been reported [30,67]. A perusal of the literature has not revealed any flavor or fragrance descriptions of the other sage oil chemotypes, however. Similarly, most bioactivity studies have been carried out on C1 chemotype sage oils. Savalev and co-workers have examined the butyryl- and acetyl-cholinesterase inhibitory activities of type C2a sage oils [56]; Lima and co-workers examined the cytotoxicity of a C2b type sage oil on rat hepatocytes [43]; and Abu-Darwish and co-workers carried out antifungal studies with chemotype C4b sage oils [14]. However, no C1c type sage oils were included in these studies for comparison. Russo and co-workers examined the cytotoxic activities on three different tumor cell lines of two different chemotypes of sage oil, C1b and C5 chemotypes, but there were no correlations between sage oil chemical compositions and cytotoxicities [54].

## 4. Conclusions

This study has revealed the presence of five major chemotypes of sage (*Salvia officinalis*) leaf essential oils, with several subtypes. Most sage oils belonged to the “typical”, α-thujone > camphor > 1,8-cineole, chemotype, but the essential oil compositions can vary widely and may have a profound effect on flavor and fragrance profiles as well as biological activities. It would be interesting to see if there exist differences in fragrance descriptions or in biological activities for the different chemotypes of sage essential oils.

## Figures and Tables

**Table 1:** Chemical compositions of leaf essential oil of *Salvia officinalis* from three different global locations.

RI ^a^	RI ^b^	Compound	Percent Composition ^c^		
			Albania ^d^	Mexico ^e^	California ^f^
847	856	(*Z*)-Salvene	0.2	0.3	0.3
855	866	(*E*)-Salvene	tr ^g^	tr	0.1
921	926	Tricyclene	0.2	0.1	0.2
926	930	α-Thujene	0.3	0.5	0.5
932	939	α-Pinene	5.0	2.4	5.2
945	954	Camphene	5.2	3.5	5.3
973	975	Sabinene	0.1	0.6	-
980	979	β-Pinene	4.1	2.6	1.3
981	979	1-Octen-3-ol	tr	0.1	0.1
989	990	Myrcene	2.8	4.5	1.2
1000	1002	α-Phellandrene	0.1	tr	-
1018	1017	α-Terpinene	0.5	tr	0.2
1022	1024	*p*-Cymene	0.6	0.2	1.3
1029	1029	Limonene	1.5	1.4	2.2
1034	1031	1,8-Cineole	26.9	15.5	11.9
1038	1037	(*Z*)-β-Ocimene	0.1	0.1	-
1042	1042	Benzene acetaldehyde	-	tr	-
1049	1050	(*E*)-β-Ocimene	-	tr	-
1059	1059	γ-Terpinene	0.7	0.7	0.4
1070	1070	*cis*-Sabinene hydrate	0.1	0.4	-
1086	1088	Terpinolene	0.2	0.2	0.3
1090	1091	*p*-Cymenene	tr	-	-
1100	1096	Linalool	0.3	tr	0.3
1103	1098	*trans*-Sabinene hydrate	-	0.4	-
1108	1102	α-Thujone	17.2	18.8	27.4
1118	1114	β-Thujone	3.8	4.4	6.0
1122	1127	Chrysanthenone	tr	-	-
1137	1138	3-*iso*-Thujanol	tr	-	-
1147	1146	Camphor	12.8	14.9	21.4
1149	1151	*neo-iso*-3-Thujanol	tr	-	-
1161	1162	*trans*-Pinocamphone	0.1	-	-
1168	1168	3-Thujanol	0.2	-	-
1169	1169	Borneol	1.2	1.0	1.7
1170	1166	δ-Terpineol	0.4	0.2	-
1180	1177	Terpinen-4-ol	0.5	0.6	0.4
1186	1188	α-Terpineol	1.1	0.4	0.4
1236	1237	Ascaridole	-	0.2	-
1254	1257	Linalyl acetate	0.2	-	-
1286	1288	Bornyl acetate	1.1	0.5	1.8
1294	1290	*trans*-Sabinyl acetate	0.1	tr	0.2
1337	1320	2,3-Pinanediol	tr	-	-
1346	1249	α-Terpinyl acetate	0.6	-	-
1375	1376	α-Copaene	0.1	-	-
1419	1419	β-Caryophyllene	4.9	3.4	3.5
1432	---	6-Oxobornyl acetate	tr	-	-
1434	1433	α-Maaliene	0.1	-	-
1439	1441	Aromadendrene	0.4	0.2	-
1446	1444	Myltayl-4(12)-ene	tr	-	-
1448	---	5-Oxobornyl acetate	0.1	-	-
1453	1454	α-Humulene	3.1	5.7	4.4
1460	1460	*allo*-Aromadendrene	-	0.1	0.1
1467	1466	9-*epi*-β-Caryophyllene	0.1	-	-
1476	1476	*trans*-Cadina 1(6)-4-diene	0.1	-	-
1482	1485	Germacrene D	-	0.1	-
1487	1483	Guaia-1(10)-11-diene	0.1	-	-
1496	1496	Viridiflorene	0.3	-	0.2
1497	1500	Bicyclogermacrene	-	0.1	-
1511	1523	δ-Amorphene	0.1	-	-
1517	1523	δ-Cadinene	0.1	-	-
1579	1578	Spathulenol	-	0.1	-
1583	1583	Caryophyllene oxide	0.1	0.2	-
1591	1592	Viridiflorol	2.0	7.4	1.5
1609	1608	Humulene epoxide II	0.2	0.3	0.2
1636	1640	Caryophylla-4(12),8(13)-dien-5α-ol	0.1	-	-
2056	2057	Manool	0.2	8.2	-
		Monoterpene Hydrocarbons	21.5	17.0	18.5
		Oxygenated Monoterpenoids	66.5	57.3	71.5
		Sesquiterpene Hydrocarbons	9.4	9.5	8.2
		Oxygenated Sesquiterpenoids	2.4	8.0	1.7
		Others	0.2	8.2	0.1
		Total Identified	100	100	100

^a^ RI = Retention index determined with respect to a homologous series of *n*-alkanes on a HP-5ms column. ^b^ Literature [13] Retention indices. ^c^ Percent composition based on peak integration without standardization. ^d^ Commercial sage leaf oil (Selikaj Ltd., Koplik, Albania). ^e^ Leaf essential oil from fresh sage (Jacobs Farm, Pescadero, California, grown in Mexico). ^f^ Commercial sage leaf oil (Mountain Rose Herbs, Eugene, Oregon, oil from California). ^g^ tr = trace (<0.05%).
